# Compensatory mechanisms in genetic models of neurodegeneration: are the mice better than humans?

**DOI:** 10.3389/fncel.2015.00056

**Published:** 2015-03-06

**Authors:** Grzegorz Kreiner

**Affiliations:** Department of Brain Biochemistry, Institute of Pharmacology, Polish Academy of SciencesKraków, Poland

**Keywords:** neurodegeneration, compensation, Alzheimer’s disease, Parkinson’s disease, Huntington’s disease, transgenic mice, genetic models

## Abstract

Neurodegenerative diseases are one of the main causes of mental and physical disabilities. Neurodegeneration has been estimated to begin many years before the first clinical symptoms manifest, and even a prompt diagnosis at this stage provides very little advantage for a more effective treatment as the currently available pharmacotherapies are based on disease symptomatology. The etiology of the majority of neurodegenerative diseases remains unknown, and even for those diseases caused by identified genetic mutations, the direct pathways from gene alteration to final cell death have not yet been fully elucidated. Advancements in genetic engineering have provided many transgenic mice that are used as an alternative to pharmacological models of neurodegenerative diseases. Surprisingly, even the models reiterating the same causative mutations do not fully recapitulate the inevitable neuronal loss, and some fail to even show phenotypic alterations, which suggests the possible existence of compensatory mechanisms. A better evaluation of these mechanisms may not only help us to explain why neurodegenerative diseases are mostly late-onset disorders in humans but may also provide new markers and targets for novel strategies designed to extend neuronal function and survival. The aim of this mini-review is to draw attention to this under-explored field in which investigations may reasonably contribute to unveiling hidden reserves in the organism.

## Introduction

The prevalence of neurodegenerative diseases, currently one of the main causes of mental and physical disabilities, has consistently risen because of the progressive aging of the worldwide population and is especially affecting highly developed societies. Examples of the more well-known neurodegenerative diseases include Alzheimer’s disease (AD), Parkinson’s disease (PD) and Huntington’s disease (HD), but a myriad of other rare neurodegenerative disorders exist, e.g., Pick’s disease, Creutzfeldt-Jakob disease (CJD), progressive supranuclear palsy (PSP), and amyotrophic lateral sclerosis (ALS). However, regardless of the nature of the disease, neural loss in the majority of the cases is estimated to usually begin 10–20 years before the first clinical symptoms appear and even a prompt diagnosis at this stage provides very little advantage for further effective treatment. Moreover, most of the cases have a sporadic occurrence, and even for those in which the genetic factors have been determined, the distinct molecular pathways leading to final cell death remain unclear. Therefore, the currently available pharmacotherapies are based on disease symptomatology and, apart from alleviating the typical symptoms, they do not restore neuronal function or prevent neuronal loss.

Most of the classic animal models for neurodegeneration are based on applying neurotoxins—an effective strategy for studying phenotype but this generates immediate neuronal death, which severely limits the opportunity to observe the molecular changes associated with the authentic, slow neurodegenerative process. Thus, the statement, “the lack of a good animal model is frustrating in efforts to curb disease progression” (Beal, 2010) appears to still be valid, despite the progress in research focused on neurodegeneration.

## Why Can We Not Fully Replicate Genetic Diseases in Transgenic Animals?

Advancements in genetic engineering over the last two decades have provided many transgenic mice that have been exploited as alternative, genetic models for various neurodegenerative diseases. These transgenic mice were created by either precisely targeting the same causative genes involved in the human disorders (e.g., HD, some rare familiar forms of PD and AD) or the genes controlling the subcellular changes and processes affected in the diverse neuropathological conditions, such as oxidative stress, rRNA synthesis, inflammation or mitochondrial dysfunction (Schwab et al., 2010; Parlato and Kreiner, 2013; Pickrell et al., 2013; Ribeiro et al., 2013). Surprisingly, many of these models do not fully recapitulate the inevitable neuronal loss (or at least not to the expected extent), supporting the proposal that different genetic, cellular and environmental factors may contribute to the ultimate cell death. Some transgenic mice fail to even demonstrate the phenotypic alterations associated with the modeled diseases, providing further evidence that humans and primates can be more vulnerable than rodents to the same triggers inducing neurodegeneration, a phenomenon also observed in pharmacological models (Przedborski et al., 2001).

In particular, extensively studied transgenic AD models, such as mice overexpressing β-amyloid precursor protein (APP, disputably but generally accepted contributing factor to AD), PS1 and PS2 (expressing mutated presenilin-1 and presenilin-2, respectively), APP/PS1 (harboring human transgenes for both APP and PS1 together) and Tg2676 (overexpressing a mutant form of APP), do not demonstrate the expected loss of neural cells (Duff et al., 1996; Oyama et al., 1998; Elder et al., 2010). Conversely, there are examples of models in which cognitive functions remain intact despite overexpression of APP (Masliah et al., 2001). Moreover, the so called “tau pathology”—formation of neurofibrillary tangles (NFT) due to hyper-phosphorylation of a microtubule-associated protein, which is another characteristic feature of AD—was not observed in most of the APP overexpressing models (Ribeiro et al., 2013). Another approach of creating AD transgenic mice models was related to tau proteins. Hyperphosphorylation of microtubule-associated protein tau (MAPT) can result in the self-assembly of NFT being involved in the pathogenesis of AD. The first transgenic model designed upon targeting tau protein did come out with any visible neurological phenotype (Gotz et al., 1995). Further attempts revealed only minor motoric impairments and tau protein accumulation (mostly in brain and spinal cord), however classic NFT were not observed or narrowed only to certain neural tissues (Eriksen and Janus, 2007; Wiedlocha et al., 2012). On the other hand, in the Htau mice characterized by expressing six isoforms of human tau without containing any mouse tau protein, the development of NFT was not correlated to the direct phenotype of the mutation and extensive neuronal loss in aged mice (Andorfer et al., 2003, 2005) indicating that the mechanism of Tau-mediated neuronal cell death remains elusive (Andorfer et al., 2005).

These discrepancies between the expected and observed phenotypes were particularly surprising in transgenic models created by directly targeting the identified causative genes. In the case of PD, there are currently recognized up to 20 genetic loci that have been described and implicated in the pathogenesis of PD, in which the mutations contribute to the familial form of PD (Scholz et al., 2012). However, none of the rodent models created by targeting these genes demonstrate profound neurodegeneration of dopaminergic cells, including the dominant mutation in leucine-rich repeat kinase 2 (LRRK2), the most widespread mutation among humans (Chesselet and Richter, 2011; Bezard et al., 2013).

Perhaps the most disappointing results have come from the classic models of HD. Huntington’s disease is a progressive autosomal dominant inherited neurodegenerative disease that is characterized by uncontrolled movements (chorea) together with emotional and cognitive symptoms and inevitably leads to death within approximately 20 years. The cause of HD has been known since 1993, when it was identified as a polyglutamine (polyQ) expansion of a stretch of CAG repeats in the amino-terminal region of the huntingtin (HTT) protein (MacDonald, 1993). Therefore, one would expect that an accurate replication of the genetic malfunction directly responsible for HD in humans should result in exactly the same phenotype in mice. In fact, knock-in HD mice with expanded polyQ tracts do not differ from their control littermates in life span or body weight, demonstrating only mild motor deficits and very moderate cell loss (Lin et al., 2001). Moreover, this resistance to the mutation is emphasized by the fact that these mouse models harbor a considerably larger CAG expansion than is necessary to induce the human form of HD. Thus, it may be concluded that mutant HTT (mHTT) appears to be more toxic to primates than to rodent models. Paradoxically, despite knowing the precise mutation responsible for HD for more than 20 years, the most studied transgenic animal model of HD is the R6/2 mouse, which was created by expressing the amino-terminal region of HTT, thus not accurately representing the cause of the disease (Li et al., 2005).

The aforementioned observations provide clues to the potential compensatory mechanisms that protect neurons from death in the evaluated genetic models, which may help us to understand the preclinical deficits observed in neurodegenerative diseases and provide better insight into the pathogenic mechanisms underlying the initial, symptomless phase of their onset. There are several candidate pathways and molecules whose activation can be considered as an effect of the compensatory processes evoked in response to the introduced mutations. The limited content of this mini-review will not allow for a discussion of all the possible dilemmas but will concentrate on a selected intriguing examples based on the very recent literature, listed in alphabetical order.

## Autophagy

Autophagy is a self-degradative process that removes unnecessary or dysfunctional cellular components through the actions of lysosomes and is important for balancing sources of energy at critical times during development, and in response to any type of cellular stress. It is generally considered a pro-survival mechanism (Glick et al., 2010). Thus, autophagy may be an essential factor that maintains neuronal homeostasis, and its impairment has been implicated in the development of neurodegenerative pathologies (Bakhoum et al., 2014). Additionally, it has also been proposed that the autophagy-lysosomal activities may play a pivotal role in neurodegenerative diseases by removing damaged or dysfunctional proteins and organelles that are a cause of oxidative stress, such that autophagy may be considered an antioxidant system (Giordano et al., 2013). Induction of autophagy has also been reported to be associated with rescue of the tau pathology, which suggests that formation of autophagosomes may be considered a compensatory mechanism rather than a trigger for neurodegeneration (Bakhoum et al., 2014). Our recent study, exploiting the new model of HD-like neurodegeneration based on the selective removal of transcription factor TIF-IA from medial spiny neurons (MSN), revealed that the transient upregulation of phosphatase and tensin homolog deleted on chromosome 10 (PTEN) kinase, a tumor suppressor that inhibits mammalian targeting of rapamycin signaling and induces autophagy, may result in enhanced MSN resistance (Kreiner et al., 2013).

## ERK (Extracellular Signal-regulated Kinases) Pathway

Alterations in the ERK pathway have been reported in some neurodegenerative diseases, accompanied by an increase in the protein levels of ribosomal S6 kinases (RSK) in several models of HD (Xifro et al., 2011). RSK are involved in cell growth and survival, and are regulated by phosphorylation controlled by mitogen-activated protein (MAP) kinases, including ERK (Chen et al., 1992). Pharmacological inhibition of RSK, as well as knock-down and overexpression experiments, have indicated that RSK activity exerts a protective effect and may act as a compensatory mechanism with the capacity to prevent cell death in HD (Xifro et al., 2011). Another study reported a role of the regulator of G-protein signaling 2 (RGS2) in controlling the compensatory response in the striatal neurons of HD models, suggesting that RGS2 inhibition may be considered an innovative target for neuroprotection (Seredenina et al., 2011). An investigation of the possible mechanism underlying RGS2-mediated neuroprotection revealed that RGS2 downregulation enhanced activation of the ERK pathway (Seredenina et al., 2011).

## Glycolysis

Glycolysis, the well-known metabolic pathway of glucose degradation resulting in formation of the high-energy compounds ATP and NADH, may also be involved in a compensatory response in neurodegenerative diseases. Glycolysis has been shown to compensate for mitochondrial dysfunction at the motor terminals of SOD1 transgenic mice (most widely used animal model of ALS), and this mechanism may help to support metabolism in the presence of dysfunctional mitochondria (Carrasco et al., 2012).

## Metalloproteases

Metalloproteases are a group of enzymes that contain a catalytic metal ion at their active site and assist in the hydrolysis of peptides, which ultimately leads to protein degradation. Metalloproteases are important in many developmental processes, including cell proliferation, differentiation and migration (Chang and Werb, 2001). Endogenous metalloproteases have been proposed to regulate mitochondrial activity in degenerating neurons, and their activation may be regarded as an adaptive and compensatory response to stressful stimuli to protect mitochondrial function (de Oca Balderas et al., 2013). Specifically, it has been shown that metalloprotease inhibition stimulates mitochondrial activity impairment induced by 3-nitropropionic acid (3-NP, striatal cell neurotoxin), and metalloproteases may be involved in the cellular reorganization induced by 3-NP (de Oca Balderas et al., 2013).

## Neurotrophins and Neurogenesis

Neurotrophins are a family of proteins that stimulate the development, differentiation and survival of neurons and are thus natural candidates for self-defense in case of neuronal loss. Neurotrophins can help to stimulate and control de novo neurogenesis. However it has been debated whether this phenomenon has any functional relevance, neurogenesis in the adult brain has also been proposed as a possible compensatory mechanism activated in response to environmental and genetic cues (Pierce and Xu, 2010).

One of the most studied and well-known triggers of neurogenesis is brain-derived neurotrophic factor (BDNF). It has been suggested that an increase in BDNF expression may reflect a compensatory mechanism against early neurodegeneration (Faria et al., 2014). Specifically, increased BDNF levels have been reported in early stages of AD; these levels decrease over the course of the disease and are inversely correlated with dementia (Laske et al., 2006). It has also been reported that serum levels of BDNF are significantly lower in PD patients and correlate with the advancement of motor impairment (Scalzo et al., 2010). Several studies have shown that a loss of BDNF protein in the brains of HD patients may contribute to the clinical manifestation of the disease (Zuccato and Cattaneo, 2014).

Turning back to animal models, it was recently shown that neurotrophin treatments used on transgenic mouse models of AD lead to a reduction in Aβ generation that was mostly dependent on the BDNF-mediated decrease in glycogen synthase kinase-3-β (GSK3β) activity, emphasizing the potential of neurotrophins as targets for disease modifying therapy (Kazim et al., 2014). Experiments performed on mice hypomorphic for TrkB tyrosine kinase receptor (mainly activated by BDNF) have revealed a profound loss of dopaminergic neurons in the region of the substantia nigra (SN) together with elevated levels of dopamine in the striatum and yet no alteration in the turnover of this neurotransmitter (Zaman et al., 2004). These findings were associated with increased BDNF levels in the striatum but not the SN, suggesting the existence of a putative compensatory mechanism that follows dopaminergic cell loss in the SN (Zaman et al., 2004). An interesting recent study by the Minichiello group in a novel genetic mouse model showed that the selective removal of BDNF from enkephalinergic striatal neurons results in spontaneous and drug-induced hyperlocomotion associated with dopamine D2 receptor-dependent increased striatal protein kinase C (PKC) and MAP kinase activation, a mechanism that may have impact on striatal neuron vulnerability in the early-stage of HD (Besusso et al., 2013).

## Noradrenaline

Noradrenaline (NA) is one of the most important neurotransmitter in the brain and the projections of noradrenergic neurons originating in the locus ceruleus penetrate virtually all brain structures. Degeneration of noradrenergic neurons is observed both in PD and AD to even greater extent and exacerbate the loss of dopaminergic and cholinergic neurons, respectively (Zarow et al., 2003). Experimental data indicate the important involvement of NA associated with PD brain damage i.e., the loss of NA in PD can worsen the dopamine nigrostriatal damage and, in opposite—an enhanced level of NA may have a neuroprotective effect (Srinivasan and Schmidt, 2003; Rommelfanger et al., 2004). These data prompt a statement that NA may serve as a compensatory mechanism in PD dopaminergic neurodegeneration (Rommelfanger and Weinshenker, 2007).

In a genetic mouse model of PD based on damaged mitochondrial DNA in dopaminergic neurons it was proven that NA and serotonin were increased after the dopaminergic cell loss (Pickrell et al., 2011). Recently, it has been shown that mirtazapine, an noradrenergic and serotonergic antidepressant drug, has a therapeutic potency in a classic pharmacological model of PD, 1-methyl-4-phenyl-1,2,3,6-tetrahydropyridine (MPTP) treated mice (Kadoguchi et al., 2014).

The activation of NA in human neuronal cultures and rat primary hippocampal neurons protects against neuronal amyloid toxicity by stimulating neurotrophic pathways (Counts and Mufson, 2010). In rats with the lesion of medial septum cholinergic neurons, sprouting of noradrenergic sympathetic fibers triggered by neurotrophins was noted, contributing to cholinergic reinnervation what experimentally reiterated the clinical implications of sprouting as an innate compensatory mechanism (Nelson et al., 2014).

## PKCδ (Protein Kinase Cδ)

PKC belongs to the group of enzymes that regulate the function of other proteins at the intracellular level and thus influence important cellular functions, including proliferation and apoptosis (Griner and Kazanietz, 2007). A balance between cell survival and apoptosis is crucial for avoiding neurodegeneration, and alterations in PKC activity have been associated with various neurodegenerative disorders, including AD, PD and HD. Recently, it was shown that an increase in the degradation of the PKCδ isoform is related to the compensatory pro-survival mechanism activated in response to mHTT-induced toxicity and is responsible for a delay in neuronal loss in HD (Rué et al., 2014). Consistent with this observation, overexpression of the PKCδ isoform *in vitro* enhances the negative effects of mHTT (Rué et al., 2014).

## Conclusions

Lack of a desired broad-spectrum phenotype and content validity of some transgenic mice used as models of various neurodegenerative diseases is often regarded as a caveat of further practical exploiting of these models and prompts researchers pursuing for alternative models, neglecting the ones which did not fully meet their expectations. However, this failure can be turned into a feature considering that behind the simple resistance to the introduced mutation, there may be a huge variety of compensatory processes delaying or attenuating the expected phenotype in the rodent genetic models. A better evaluation and understanding of these mechanisms may help us to not only explain why neurodegenerative diseases are mostly late-onset disorders in humans but may also provide new disease markers and targets for novel strategies designed to extend neuronal function and survival. Such attempts are apparently rare. Surprisingly, only very few studies describing the observed phenotypes of different genetic models of neurodegenerative diseases have focused on this problem, and these mechanisms were barely investigated in these papers.

The aim of this mini-review is to focus attention on this under-explored field in which investigations may reasonably contribute to unveiling hidden reserves within the organism, particularly important in a preclinical stages of neurodegenerative diseases. Perhaps this is the time to reevaluate the initial descriptive characteristics of the transgenic mice created upon targeting the same causative genes as in human neurodegenerative diseases, and pursue the potential compensatory mechanisms underlying introduced mutations that do not result in the expected phenotype.

## Conflict of Interest Statement

The author declares that the research was conducted in the absence of any commercial or financial relationships that could be construed as a potential conflict of interest.
